# An overview of the mechanistic approaches of antifungal nanomaterials

**DOI:** 10.3389/fphar.2025.1701446

**Published:** 2025-11-17

**Authors:** Sarmistha Saha, Nadezhda Sachivkina, Regina Gurina, Ekaterina Neborak, Natallia Zhabo, Marina Avdonina, Maria Molchanova

**Affiliations:** 1 Department of Biotechnology, Institute of Applied Sciences & Humanities, GLA University, Mathura, Uttar Pradesh, India; 2 Department of Microbiology V.S. Kiktenko, Institute of Medicine, Peoples’ Friendship University of Russia Named After Patrice Lumumba (RUDN University), Moscow, Russia; 3 Agrarian and Technological Institute, Peoples’ Friendship University of Russia Named After Patrice Lumumba (RUDN University), Moscow, Russia; 4 Department of Biochemistry T.T. Berezov, Institute of Medicine, Peoples’ Friendship University of Russia (RUDN University), Moscow, Russia; 5 Department of Foreign Languages, Institute of Medicine, Peoples’ Friendship University of Russia Named After Patrice Lumumba (RUDN University), Moscow, Russia; 6 Department of Linguistics and Intercultural Communication, Moscow State Linguistic University, Moscow, Russia

**Keywords:** antifungal, antibiotics, nanotechnology, antimicrobial resistance, *Candida* species

## Abstract

Antifungal medications currently available on the market have significant drawbacks, including serious side effects and poor absorption. Nanotechnology offers a promising solution to address these issues. Metal nanoparticles, polymer nanoparticles, dendrimers, liposomes, and carbon quantum dots are often employed nano-delivery systems in antifungal therapy. While nanotechnology has several benefits, including improved oral bioavailability, less side effects, controlled release, and targeted delivery, it also has significant drawbacks. We reviewed the limitations of current commercial antifungal solutions, the primary mechanistic insights by which nanotechnology can enhance antifungal efficacy, and the challenges associated with these approaches. For optimum therapeutic interventions, modifying the surfaces of nanomaterials could be considered to improve their interaction with fungal cells. This can be achieved through targeted delivery to the fungal cell wall and membrane or by utilizing electrostatic interactions, which allow nanoparticles to effectively adhere to fungal cells. Additionally, custom-designed nanomaterials can overcome challenges posed by physiological barriers such as the blood-brain barrier, corneal barrier, and skin barrier. Despite the challenges of implementing nanotechnology in antifungal treatments, its potential and innovative applications open up new possibilities for effective antifungal therapies in the future.

## 1 Introduction

The first Fungal Pathogen Priority List (FPPL), released by the World Health Organization (WHO) at the end of 2022, identified 19 fungi as serious dangers to public health worldwide, including *Candida auris, Aspergillus fumigatus, Cryptococcus neoformans*, and *Candida albicans*. Each year, an estimated 1.6 million people succumb to fungal infections, which affect over 1 billion individuals worldwide (Tufa and Denning, 2019; Brown et al., 2012). These pathogens are linked to more than 60,000 deaths and 100 million illnesses annually (Rokas, 2022; Du et al., 2021). Understanding the diverse range of fungal infections is crucial, as they can arise from indigenous, opportunistic, or external pathogenic species (Garibotto et al., 2010; Wu et al., 2023). Moreover, weakened immune systems such as those living with HIV/AIDS, severe combined immunodeficiency (SCID), certain endocrine metabolic diseases, or those who have undergone organ transplants are at increased risk for developing invasive fungal infections (Zhu et al., 2023). This highlights the importance of early detection and intervention in vulnerable populations. Each year, an estimated 3.8 million people succumb to IFIs, affecting over 6.5 million individuals worldwide (Denning, 2024). The primary pathogens responsible for these infections include *Fusarium* spp., *Aspergillus* spp., *Candida* spp., and *Cryptococcus* spp (Bongomin et al., 2017).

Even while antifungal medications now on the market have shown great effectiveness in treating both superficial and invasive fungal infections, using them is frequently linked to drawbacks and restrictions. For instance, allylamines are mostly used to treat superficial fungal infections (Crawford and Hollis, 2007), and azole resistance is on the rise (Mohd-Assaad et al., 2016; Mohr et al., 2008). Polyenes may also result in responses associated to infusion (Roemer and Krysan, 2014). Furthermore, the complicated intravenous administrations and related toxicities of these medications sometimes restrict their clinical use for invasive infections (Campoy and Adrio, 2017). The problem of treatment is exacerbated by the emergence of fungi that are resistant to both single and multiple drugs. This includes species such as *Candida glabrata*, which is resistant to echinocandins, and *Candida auris*, which is resistant to multiple drug classes including azoles, polyenes, and echinocandins. Additionally, resistance to azoles is increasing in both *Aspergillus* and *Candida* species (Perlin et al., 2017). The growing rates of resistance exacerbate the small chemical space and target sites that are blocked by existing antifungals. The formation of efflux pumps, decreased expression of drug targets or structural changes to drug targets, and biofilm formation are some of the mechanisms underlying antifungal resistance (Gupta and Xie, 2018). These processes can be learned by vulnerable species, but they are intrinsic in fungal species like *C. auris* that are less sensitive to antifungal medications.

Researchers are looking into the possible uses of nanotechnology in antifungal drug delivery in addition to investigating novel antifungal drug species (Gupta and Xie, 2018). Through a variety of mechanisms, including compromising the integrity of the fungal membrane through charge interactions, encouraging the production of reactive oxygen species (ROS), and changing the permeability of fungal cell membranes, nanosystem-based drug delivery can produce high local drug concentrations at the targeted site, facilitating antifungal effects (Mu and Feng, 2003; Peer et al., 2007). Drug delivery nanosystems have therefore become the best way to deliver drugs (Erdogar et al., 2018).

The study on antifungal nanosystems is now covered in a number of reviews. The use of nanosystems to treat particular fungal infections has been compiled by some researchers (Negi et al., 2023; Araujo et al., 2020). Furthermore, other researchers have presented different kinds of nanosystem for antifungal treatment (Nami et al., 2021; Voltan et al., 2016). These evaluations do not, however, offer a comprehensive overview of the mechanisms targeted by different antifungal nanosystems; rather, their primary goal is to introduce distinct nanoparticle kinds. There is currently no thorough review devoted exclusively to the mechanisms of various antifungal nanosystems, despite the fact that some researchers have given overviews of nanosystems for antifungal drug delivery. However, this review study offers a fresh viewpoint on using nanotechnology to fight fungal diseases by highlighting three crucial mechanisms to improve antifungal efficacy. The use of nanotechnology in antifungal medicine will be thoroughly examined in this paper, with an emphasis on improving interaction with fungus.

## 2 Antifungal antibiotics and their challenges

Antifungal medications present a unique challenge due to the similarities in cellular structure and metabolic processes between fungal and human cells, both of which are eukaryotic. This commonality often results in significant cytotoxicity, leading to a more limited range of antifungal options compared to antibacterial agents. Despite these challenges, there is potential for growth in this field, as the need for new and effective antifungal treatments remains critical. Currently, clinicians utilize four main types of antifungal medications to combat fungal infections: polyenes, echinocandins and azoles (Fisher et al., 2022; Mota Fernandes et al., 2021).

**FIGURE 1:**
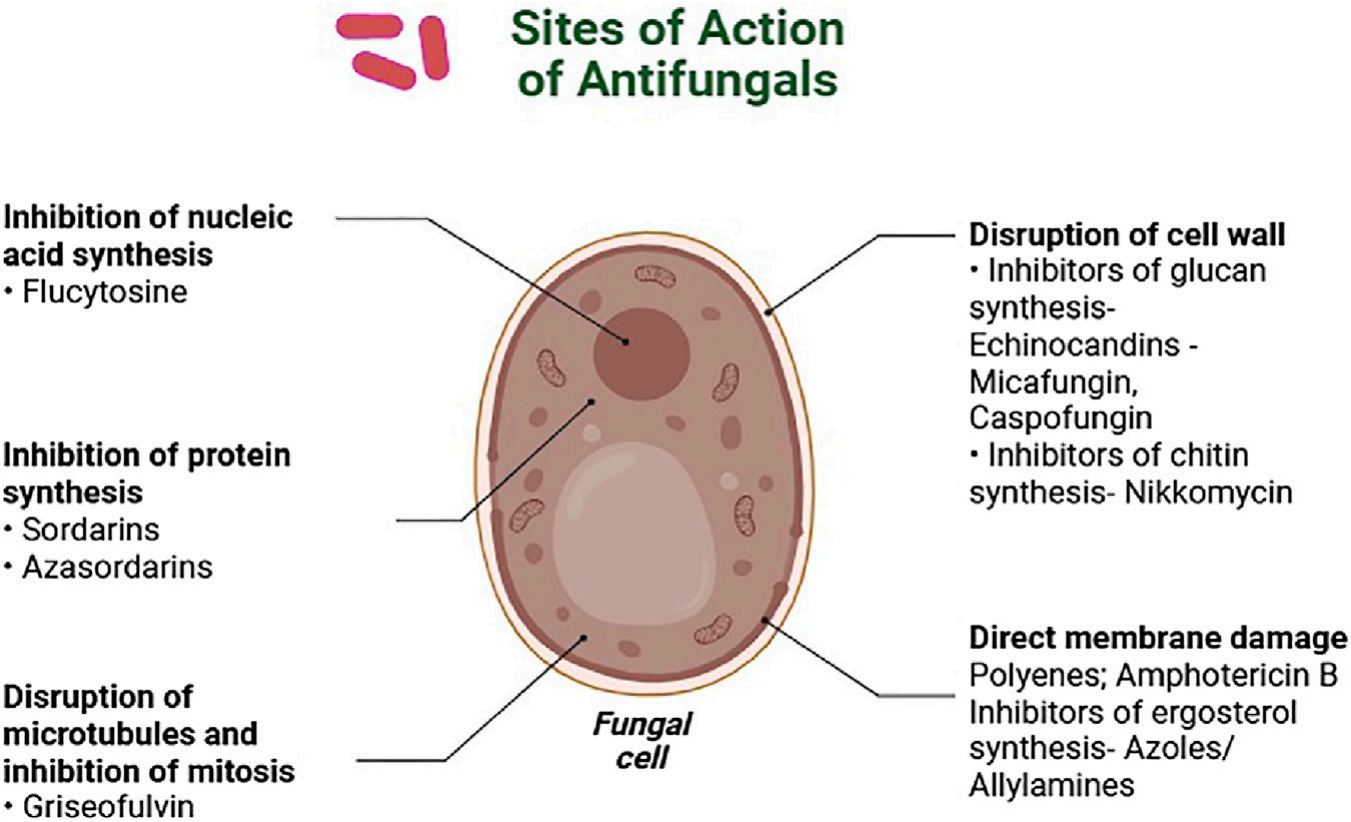
Antifungal antibiotics and their sites of action.

Polyene medications, particularly Amphotericin B (AmB) and other polyene antifungals have shown high potency against serious fungal infections by binding to ergosterol in the cell membrane (Figure 1) (Carolus et al., 2020). Given its strong antifungal properties and broad spectrum of activity, AmB is frequently utilized to address severe fungal conditions, including invasive candidiasis, aspergillosis, and cryptococcosis. While the effectiveness of AmB is noteworthy, its use can be limited by significant toxicity. To enhance patient safety and minimize these adverse effects, advancements in formulation technology have led to the development of AmB-containing polymers and liposomal formulations. These include liposomal AmB (AmBisome), AmB-lipid complex (ABLC), and AmB colloidal dispersion (ABCD), which can provide effective treatment options while reducing the associated risks (Lewis, 2010).

Azole antifungal medications play a vital role in combating fungal infections. They achieve this by inhibiting the enzyme lanosterol 14α-demethylase, which is necessary for the production of ergosterol, a critical component of the fungal cell membrane (Lewis et al., 2022). By disrupting the synthesis of ergosterol, azoles can effectively weaken the fungal cell membrane, impacting the fungus’s growth and reproductive capabilities. These medications are commonly prescribed for a range of conditions, including nail fungus, aspergillosis, cutaneous mycoses, and *Candida* infections, such as candidemia. It is important to be aware that while azoles are highly effective, they may also lead to side effects, including headaches, gastrointestinal discomfort, and potential liver damage (Como Jackson and Dismukes William, 1994).

Echinocandins represent an innovative class of antifungal treatments, including micafungin, caspofungin, and anidulafungin. These agents work by targeting the enzyme β-1,3-D-glucan synthase, which is essential for building fungal cell walls (Chen et al., 2011). By inhibiting this enzyme, echinocandins can promote fungal cell death due to the formation of inadequate cell walls. Echinocandins target 1,3-β-glucan synthesis and their targeted approach makes them a valuable addition to antifungal therapy (Hashimoto, 2009).

Pyrimidine analogs, such as 5-fluorocytosine, play a vital role in disrupting DNA and RNA synthesis by imitating the structure of pyrimidine (Coelho et al., 2024). This mechanism effectively hinders the replication of genetic material and the formation of proteins in fungal cells. Fluorocytosine is particularly valuable in the treatment of cryptococcosis and candidiasis. Its combination with other antifungal medications can enhance therapeutic outcomes and maximize effectiveness. However, it is important to monitor for possible side effects, including hepatotoxicity and bone marrow suppression, as well as to be aware of the potential for developing resistance (Vermes et al., 2000).

Addressing fungal infections involves navigating several challenges, but the current landscape also presents opportunities for innovation and improvement in treatment strategies. One primary challenge is the limited number of antifungal medications available, which often target a single therapeutic pathway. However, this limitation also encourages the development of new therapies that could offer broader and more effective options. One significant obstacle to effective treatment is the emergence of drug-resistant fungal strains. While the biofilms can complicate drug treatment efforts, it also highlights the need for innovative approaches to overcome these barriers (Fisher et al., 2022).

Furthermore, strong antifungal agents, like Amphotericin B (AmB), are frequently associated with toxic side effects. Although these side effects can limit treatment duration and impact patients’ quality of life, they underscore the importance of advancing research and developing new formulations or alternatives that minimize risks (Gavaldà et al., 2005).

In light of these challenges, the search for new antifungal agents and treatment methods, particularly those that employ novel mechanisms of action, is gaining momentum. In this context, nanotechnology approach not only holds the potential to improve treatment efficacy but also opens new avenues for combating stubborn fungal infections.

## 3 Antifungal nanostructures based on their mechanism

### 3.1 Cell-mimicking techniques

**FIGURE 2:**
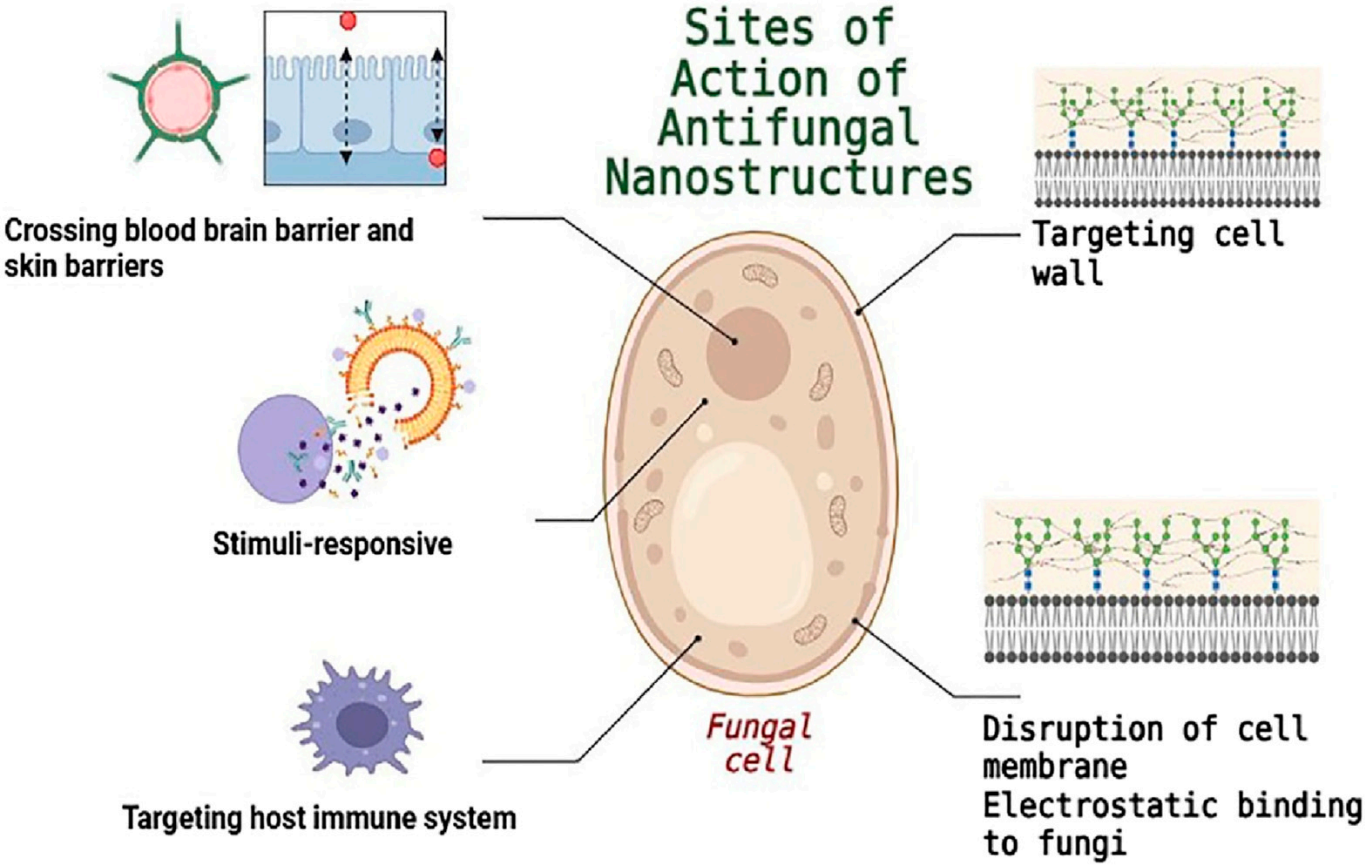
Mechanisms of using nanomaterial-based strategies to enhance antifungal therapy.

Recent innovations in biomimetics have significantly advanced the use of cell membrane camouflage as a strategic approach in therapeutic applications. One noteworthy development by Lin et al. (2023) is the creation of a biomimetic nanoplatform, designated VM(IR780)-PFC(O2). This “three-in-one” platform incorporates nanoparticles coated with vaginal epithelial cell membranes alongside a photosensitizer which selectively targets *C. albicans* (Figure 2). Moreover, the formation of pores on the surface of the nanoplatform accelerates the release of both the photosensitizer and oxygen after candidalysin sequestration, thereby enhancing antifungal efficacy upon exposure to near-infrared radiation (Lin et al., 2023). Using this nanoplatform to treat an intravaginal *C. albicans*-infected mouse model results in a marked reduction in the burden of *C. albicans*, especially when using candidalysin to further increase phototherapy and inhibit *C. albicans*.

Addressing the complexities of membrane characteristics, an innovative “molecular affinity” technique was introduced to ensure that cell membrane-modified nanocarriers maintain proper orientation and coating on nanoparticulate cores. By utilizing peptide ligands derived from the cytoplasmic protein P4.2, they successfully modified the surfaces of cationic liposomes. This interaction facilitates the formation of a “hidden peptide button,” promoting the desired “right-side-out” orientation during the coating process. As a result, liposomes covered with cell membranes are particularly effective in targeting *C. albicans*, thus neutralizing the fungal hemotoxins (Xie et al., 2019).

Furthermore, immune cells exhibit strong binding capacities with fungi, enhancing the potential of using therapeutic nanoparticles coated with macrophage membranes, which can specifically target and eradicate fungal cells with great efficacy (Han et al., 2022; Chen et al., 2024). For example, research has demonstrated that macrophage cell membranes can encapsulate lanthanide-doped upconversion nanoparticles (UCNPs) integrated with DNA sensing elements and the photosensitizer methylene blue. This formulation showcases remarkable functionalities, including cytokine isolation, fungal diagnostics, antifungal adherence, and targeted fungal elimination (Table 1). The impressive cell membrane camouflage not only boosts the fungicidal efficacy of UCNPs but also stabilizes the particles and enhances their biocompatibility, highlighting their promising applications in photodynamic therapy, wound healing enhancement, and fungal detection (Wang et al., 2024).

**TABLE 1:** Overview of the antifungal potentials of nanomaterials based on their mechanisms.

Antifungal nanocomposite	Mechanisms of action	Tested fungal strains	References
Phototherapy using vaginal epithelial cell membrane	Cell-mimicking	*C. albicans*	Lin et al. (2023)
Upconversion nanoparticles with macrophage membranes	Cell-mimicking	*C. albicans*	Wang et al. (2024)
Silver nanoparticles	Electrostatic attachment	*C. glabrata* biofilms	Monteiro et al. (2012)
AgNPs	Electrostatic attachment	*C. albicans*, *C. glabrata* and *C. tropicalis*	Rahisuddin et al. (2015)
Copper nanoparticles (CuNPs)	Electrostatic attachment	*Corticium salmonicolor*	Cao et al. (2014)
Iron oxide nanoparticles	Electrostatic attachment	*C. albicans* biofilms	Golipour et al. (2019)
Zinc 2-methylimidazolate frameworks	Electrostatic attachment	*C. albicans*	Su et al. (2020)
Ketoconazole–chitosan–gellan gum	Electrostatic attachment	*A. niger*	Kumar et al. (2016)
Chitosan nanoparticles	Electrostatic attachment	*C. albicans*	Panwar et al. (2016)
Quaternary trimethyl chitosan–silver nanoparticles	Electrostatic attachment	*S. rolfsii* and *F. oxysporum*	Alli et al. (2024)
Polyethylene glycol (PEG)-imidazole modified G5 dendrimer	Electrostatic attachment	*A. fumigatus*	Tang et al. (2021)
Amphotericin-loaded liposomes with monomers of Dectin-2’s mannan-binding domain	Targeting fungal cell wall	*C. albicans*, *C. neoformans*, and *A. fumigatus*	Ambati et al. (2019)
Poly(lactic-co-glycolic acid) (PLGA) nanoparticles conjugated with chitosan-binding peptide	Targeting fungal cell wall	*C. neoformans*	Tang et al. (2018)
N-Succinyl chitosan/gold nanocomposites	Targeting cell membrane	*C. albicans*	Dananjaya et al. (2020)
Gold nanodiscs	Targeting cell membrane	*C. albicans*	Wani and Ahmad (2013)
CuO nanoparticle composites with polylysine–alginate nanogels	Targeting fungal cell membrane	*A. alternata*	Zhu et al. (2022)
Copper sulfide (CuS) nanoparticles	Penetrate the fungal cell wall	*C. albicans*	Zhu et al. (2023)
Carbon dots	Penetrate the corneal barrier by opening the tight junctions	*Fusarium solani*	Chen et al. (2024)
Poly(butyl cyanoacrylate) polymeric nanocapsules	Penetrate the blood-brain barrier	*Cryptococcus* spp.	Ćurić et al. (2017)
Multilamellar vesicles coated with opalmitoylated mannan	Targeting macrophages		Vyas et al. (2000)
Mannosylated chitosan oligosaccharides-based nanoparticles	Targeting macrophages	*C. albicans*	Gao et al. (2020)
Glycol chitosan nanoparticles	ROS-responsive	*Fusarium* and *Aspergillus flavus*	Niu et al. (2023)
Poly(salicylic acid) (PSA) co-assembled nanoparticles	pH-responsive	*Sclerotinia sclerotiorum and Fusarium graminearum*	Huang et al. (2022)
Block-copolymers of P(PEGMA-b-DEAEMA) nanoparticles	pH-responsive	*C. albicans*	Albayaty et al. (2020)
Albumin nanoparticles conjugated with BSA-binding peptide and an MMP-3-responsive peptide	Targeting matrix metalloproteinase 3	*C. neoformans*	Cheng et al. (2021)
PEG-PCL polymers nanoparticles	Lipase-responsive	azole-resistant** ** *C. albicans*	Yang et al. (2019)
Reduced graphene oxide (rGO)] with *Lactobacillus* and hyaluronic acid-based hydrogels	H_2_O_2_-responsive	*C. albicans*	Wei et al. (2023)
Lyticase and gallium ions co-integrated nanosystems	Interfere with antioxidant-, exopolysaccharide-, iron-ion-utilization-, biofilm-development-, and virulence- related genes	*C. albicans*	He et al. (2022)
(NIR) laser-propelled parachute-like nanomotor	Photothermal effects	*C. albicans*	Ji et al. (2021)
NIR responsive conjugated polymer nanoparticles	Photothermal damage, activates calcium-calmodulin signaling followed by the LC3-associated phagocytosis	*A. fumigatus*	Wang et al. (2023)
Copper-proanthocyanidins** **nanozyme-thixotropic anionic hydrogel	Scavenging of ROS through the catalase-like and superoxide dismutase-like activities	*F. solani*	Shi et al. (2024)
Hypocrellin A conjugated with a polyhedron and polyethylene glycol	Chromatin reorganization	*C. auris*	Liu et al. (2025)

### 3.2 Electrostatic attachment to fungus

Several techniques were created to improve the treatment through ionic interactions since fungal cell walls and membranes are often negatively charged (Figure 2) (Ye et al., 2022). Metallic nanoparticles, recognized for their potent fungicidal properties, represent a promising area of research. Monteiro et al. synthesized silver nanoparticles (AgNPs) of various sizes using two distinct stabilizers: polyvinylpyrrolidone and ammonia (Monteiro et al., 2012). Their research revealed that the minimum inhibitory concentration (MIC) values for *C. glabrata* ranged between 0.8 and 3.3 μg/mL, while those for *C. albicans* fell between 0.4 and 0.8 μg/mL, indicating a consistent response. When it came to quantifying biomass, AgNPs were only highly efficient against *C. glabrata* biofilms, reducing biomass by almost 90% at a concentration of 108 μg mL^−1^ (Monteiro et al., 2012). The researchers thoughtfully proposed that the observed findings could be attributed to particle aggregation, as it is likely that the nanoparticles first gathered on the surfaces of the microorganisms before exhibiting their antibacterial effects. According to reports, AgNPs are more effective than many antifungal medications, against *C. albicans* (Table 1) (Rahisuddin et al., 2015; Mallmann et al., 2015), *C. tropicalis* (Mallmann et al., 2015), and *S. cerevisiae*. Ag^+^ ions emitted from the AgNPs may also have antifungal properties in addition to the AgNPs’ inherent antifungal action (Li et al., 2013). TEM pictures showed that AgNPs were heavily accumulated outside of the cells. Instead of penetrating the cells, the authors hypothesized that the AgNPs employed in this work produced Ag^+^ ions, which entered the cells and caused NPs to form by chemical reduction by organic compounds found in the cytoplasm and cell wall (Vazquez-Muñoz et al., 2014). In comparison to AgNPs, the potential use of gold nanoparticles (AuNPs) for antifungal applications has garnered significantly less experimental attention. One study found that the MIC of 25 nm and 30 nm AuNPs against *Candida* species was between 16 and 32 μg/mL and 32–128 μg/mL, respectively (Wani et al., 2013). This study produced two different sizes of AuNPs using various reducing agents. It suggests that smaller AuNPs may be more effective against fungi, a trend that has been observed in other investigations as well (Ahmad et al., 2013; Swain et al., 2016). It has been observed that copper nanoparticles (CuNPs) have antifungal properties against a wide variety of fungus species. The MIC of approximately 2.5 μg/mL was demonstrated by CuNPs (9–34 nm) produced by thermal decomposition (Wei et al., 2010), 7 μg/mL by ultrafine CuNPs (2–4 nm) produced by chemical reduction against *Corticium salmonicolor* (Cao et al., 2014), and 3.9 μg/mL by carboxymethylated chitosan-stabilized CuNPs (4–15 nm) produced by chemical reduction against *C. tropicalis* (Tantubay et al., 2015). Furthermore, CuNPs have demonstrated encouraging antifungal properties when coated on a range of polymer surfaces (Cioffi et al., 2005; Cioffi et al., 2004). Additionally, iron oxide nanoparticles had inhibitory effects on fungal biofilm formation. Specifically, at 50 μg/mL, iron oxide nanoparticles caused about 70% suppression of *C. albicans* biofilm, and at 200 μg/mL, it achieved 100% inhibition of biofilm formation (Golipour et al., 2019). Further research is necessary to completely understand the antifungal mechanisms of iron oxide nanoparticles.

Metal-Organic Frameworks (MOFs) are innovative, highly ordered porous materials created through the self-assembly of organic ligands and metal ions or clusters (Jiao et al., 2019). Their potential in tackling fungal infections has been evidenced by their capacity to improve drug delivery. For example, Su et al. (2020) demonstrated the use of voriconazole as a key component in the development of voriconazole-inbuilt zinc 2-methylimidazolate frameworks (V-ZIF). This approach effectively enhanced drug penetration efficiency by promoting strong binding to negatively charged cell membranes. The positive surface charge and optimal size of V-ZIF played a crucial role in facilitating this process. Additionally, in a mouse model, the V-ZIFs had minimal adverse effects on the healthy tissues of the main organs while eliminating open-wound infections brought on by *C. albicans* more effectively than voriconazole alone. Moreover, this design significantly enhanced the penetration of *C. albicans* biofilms by utilizing the nanoscale and surface properties of the MOFs, highlighting the potential for more effective antifungal therapies (Su et al., 2020). Furthermore, a notable study introduced a co-delivery MOF system that combined the hydrophobic fungicide fenhexamid with the plant immune inducer salicylic acid, using a Zn-Al hydrotalcite (HTlc)-like nanosheet as the carrier (Gao et al., 2024). This innovative co-delivery method not only bolstered penetration ability of drug but also effectively enhanced antifungal activity.

The non-toxic cationic polymer chitosan, produced through the N-deacetylation of chitin, shows promising antifungal effects. Its polycationic nature, particularly the protonation of its functional amino groups, facilitates electrostatic interactions with negatively charged macromolecules in cell walls (Sebti et al., 2005). This interaction significantly enhances cell membrane permeability, ultimately leading to cellular disruption and death. Additionally, strong gels can be formed using gellan gum, a biocompatible, biodegradable, and non-toxic heteropolysaccharide derived from *Pseudomonas elodea*. Research conducted by Thakur and colleagues has demonstrated the effectiveness of ketoconazole–chitosan–gellan gum (CSGG) nanoparticles against *Aspergillus niger*, while also effectively reducing the negative side effects commonly associated with ketoconazole. The electrostatic interaction between CSGG nanoparticles and fungi paves the way for improved delivery of antifungal medications, enhancing their therapeutic efficacy and minimizing adverse effects (Kumar et al., 2016). Moreover, chitosan encapsulation has been shown to increase the stability and bioavailability of various antifungal drugs. Furthermore, recent studies have shed light on the potential of ferulic acid (FA), a key phenolic compound, to inhibit biofilms of *C. albicans* (Raut et al., 2014). Despite its promise, FA faces challenges due to its instability and limited permeability (Munin and Edwards-Lévy, 2011). However, utilizing chitosan encapsulation to create ferulic acid encapsulated chitosan nanoparticles (FA-CSNPs) has led to a notable reduction in the metabolic activity of *C. albicans* by up to 22.5% during a 24-h incubation period. This is a significant improvement compared to native FA (63%) and unloaded CSNPs (88%) (Panwar et al., 2016).

The recent study on quaternary trimethyl chitosan–silver nanoparticles has shown promising results in combating plant pathogens such as *S. rolfsii* and *Fusarium oxysporum*. When exposed to these nanoparticles, *S. rolfsii* exhibited an impressive 100% growth inhibition, while *F. oxysporum* showed a substantial 76.67% reduction in growth. These nanostructures function effectively due to their dual antibacterial and antifungal properties. Their mechanism of action involves the electrostatic attachment to the fungal cells, which facilitates the rupture of cell walls and membranes. This disruption leads to the leakage of intracellular contents, ultimately contributing to fungal cell death (Alli et al., 2022).

The low water solubility and high dosage requirements of traditional antifungal medications, such as posaconazole (POS), make treatment efficacy and safety problematic. In another innovative research, 4-nitrophenyl chloroformate was effectively utilized to activate the POS derivative, N-(POS-carbonyl) diglycolamine. This activation enables further treatment with generation 5 (G5) dendrimers, facilitating the creation of indirect conjugates. The study successfully developed two classes of compounds: those directly conjugated to the activated POS and those indirectly conjugated to the activated POS derivative D. As a result, this method proved effective in inhibiting the growth of *A. fumigatus* for over 96 h (Tang et al., 2021).

### 3.3 Targeting fungal cell walls and membranes

Fungal cells are protected by a thick cell wall, which is essential to their survival and pathogenicity in contrast to mammalian cells (Kang et al., 2018). Antifungal therapy targets the fungal cell wall because of its distinct makeup, which is not present in human cells (Figure 2). A promising mammalian protein, dectin-1, effectively binds to β-glucan polysaccharides, which are prevalent in the cell walls of most fungi. By modifying liposomes with dectin-1 and loading them with amphotericin B (AmB), researchers have developed a target-specific formulation known as DEC-AmB-LL. This innovative approach not only enhances binding to fungal cells but also reduces the necessary dosage of AmB while effectively suppressing *A. fumigatus*. Remarkably, DEC-AmB-LLs demonstrate a tenfold increase in binding efficiency compared to unmodified liposomes (Ambati et al., 2019). However, it is important to note that these dectin-1-coated liposomes exhibit limited binding ability to *C. albicans*, likely due to the presence of thick mannan polysaccharide layers that obscure the β-glucans. In response to this challenge, researchers have developed DEC2-AmB-LL liposomes, which have been tailored to bind more effectively to *C. albicans*, *C. neoformans*, and *A. fumigatus*. By utilizing dectin-2, another mammalian innate immune receptor that binds mannans in a dimeric state, these modified liposomes significantly enhance binding efficiency by 50–150 times compared to their unmodified counterparts (Ambati et al., 2019).

Furthermore, many fungal cell walls contain chitosan, a deacetylated form of chitin, which represents an important target for antifungal treatments (Roy et al., 2021). The chitosan-binding peptide serves as a valuable targeting ligand that can improve nanoparticle adhesion to fungi. For instance, Tang et al. (2018) successfully identified a CP using phage display technology. They then conjugated this peptide onto poly(lactic-co-glycolic acid) (PLGA) nanoparticles used as carriers for itraconazole. When exposed to free chitosan, the nanoparticles demonstrated enhanced adhesion to mucosal layers through noncovalent binding. This improvement significantly facilitated the nanoparticles’ ability to penetrate the bloodstream and overcome the oral absorption barrier. In preclinical studies involving mouse models, the nanoparticles showed remarkable efficacy in reducing lung infections caused by *C. neoformans* (Tang et al., 2018).

Research indicates that AuNPs demonstrate promising antifungal properties through a combination of enzyme deactivation and cell membrane disruption. For example, studies have shown that fungal cell membranes sustain damage following treatment with AuNPs (Dananjaya et al., 2020). N-Succinyl chitosan/gold nanocomposite exhibited a strong antifungal activity against pathogenic *C. albicans* via destruction of *C. albicans* cell membrane (Dananjaya et al., 2020). The *in vivo* investigations further validated the nanocomposite’s inhibitory effects on *C. albicans* hyphae production in infected zebrafish muscle tissue. Additionally, gold nanodiscs may engage with the transmembrane protein H^+^-ATPase, an ATP-driven enzyme that utilizes the primary active transport of hydrogen ions (H^+^) to create electrochemical potential differences across biological membranes. This process is crucial for the growth and development of fungal cells, which depend on secondary transport mechanisms fueled by proton gradients. By binding to H^+^-ATPase, AuNPs potentially disrupt its function, affecting the metabolism of the fungus and leading to its eventual death (Wani and Ahmad, 2013).

### 3.4 Crossing biological barriers

Zhou et al. (2023) suggested a method that entailed breaking down extracellular polysaccharides, specifically β-1,3-glucan, in order to boost the sensitivity of antifungal techniques and penetrate the fungal cell wall and extracellular polysaccharide barriers. In order to break down the extracellular polysaccharides found in cell walls and biofilms, they created an integrated nanosystem that combines lyticase and Ga ions. By releasing gallium ions, this nanosystem eliminated mature biofilms and *C. albicans* (He et al., 2022). Zhao and colleagues (2023) have successfully developed a promising biodegradable microneedle patch aimed at treating deep cutaneous fungal infections. This innovative patch incorporates hyaluronic acid (HA) loaded with copper sulfide (CuS) nanoparticles and the antimicrobial peptide PAF-26. When the microneedle tips penetrate the epidermis, they gradually disintegrate, releasing PAF-26 and CuS nanoparticles, which effectively target and eliminate the fungus in a DCFI mouse model. The CuS nanoparticles play a crucial role by catalyzing hydrogen peroxide to generate reactive oxygen species (ROS) that specifically target fungi. At the same time, PAF-26 directly disrupts the envelopes of fungal cells. Once the fungal cell membranes are compromised by PAF-26, the ROS can rapidly enter the fungal cells, significantly enhancing the overall antifungal action (Wang et al., 2023).

Chen and colleagues develop a positively charged ultrasmall positively charged carbon dot that can effectively navigate through the corneal epithelium thanks to its small size and extracellular pathways (2024). This platform not only allows for easier penetration but also enhances eye retention time through electrostatic attraction. At low concentrations, these carbon dots exhibit impressive antifungal activity when taken up by *Fusarium solani* fungi, triggering the release of ROS that damage the fungal cell membranes. *In vivo* studies in female mice with fungal keratitis have confirmed that carbon dots are capable of successfully treating keratitis and eradicating fungal infections. Additionally, by temporarily loosening the tight junctions in the corneal epithelium, carbon dots show an enhanced capacity to penetrate the corneal barrier, thereby improving therapeutic effectiveness in the deeper layers of the stroma.

Using a Schiff base reaction between a self-synthesised polyaldehyde oligomer (PAO) and amino-functionalized hyaluronic acid (AHA), Shi and associates (2024) created a multi-enzyme mimicking nanozyme-thixotropic anionic hydrogel coating (NHC) to cure fungal keratitis. In reaction to changes in stress or strain, the thixotropic hydrogel may change its viscosity or flow characteristics. NHC significantly improved voriconazole’s permeability and retention duration, allowing for a low dosage to achieve therapeutically efficacious levels. Additionally, by using a HA derivative to stimulate cell proliferation, NHC aided in the repair of corneal wounds. Furthermore, CuPC’s catalase-like and superoxide dismutase-like properties helped to fight ROS, improving the therapeutic efficiency in the treatment of fungal keratitis (Shi et al., 2024).

Low-density lipoprotein receptors (LDLRs) found on the surfaces of brain capillary endothelial cells can identify apolipoprotein E (Apo E) (Kreuter, 2007). By attaching Apo E to nanoparticles, we can enhance their ability to enter the brain. This approach of using Apo E to modify nanoparticle-based drug delivery systems is an effective strategy for penetrating the blood-brain barrier (BBB). For example, Fricker and colleagues developed poly(butyl cyanoacrylate) polymeric nanoparticles (PBCA-NCAs) loaded with itraconazole. They modified the PBCA-NCAs using DSPE-PEG(2000)-maleimide and then linked these modified nanoparticles with Apo E (Ćurić et al., 2017). This modification enabled high doses of the poorly soluble itraconazole to be effectively transported across the blood-brain barrier to reach its therapeutic target.

In a very recent study, a novel photosensitizer was synthesized based on hypocrellin A (HA), modified via conjugation to a covalent organic polyhedron (COP1T) and polyethylene glycol (PEG) (Liu et al., 2025), which showed excellent antifungal activity against *Candida auris*. Their findings demonstrated that while high-dose results in more noticeable alterations in A/B compartmentalization, topologically associating domain organization, and chromatin looping linked to important genes involved in mitochondrial energy metabolism along with ROS accumulation near the nucleus, whereas, low-dose results in minor local changes in chromatin topology. The same study team also demonstrated that this approach enhanced wound healing and dramatically decreased fungus burden. It reduced systemic inflammation and promoted myeloid and type 2 innate lymphoid cell infiltration locally (Liu et al., 2025). A balanced cytokine profile that supports tissue healing and fungus removal was validated by transcriptomic data.

### 3.5 Targeting host innate immune system

The initial line of defense against fungal infections is the host’s innate immune system. In order to mediate fungal clearance, host innate immune cells use pattern recognition receptors (PRRs) to identify invasive fungi. They then start a sequence of effector mechanisms and adaptive immune responses. Vyas et al. (2000) developed amphotericin B (AmB)-encapsulated multilamellar vesicles that are coated with opalmitoylated mannan (OPM). The OPM-coated liposome formulations demonstrated superior antifungal efficacy and higher accumulation in macrophages compared to unmodified liposomes. Additionally, another study explored the interaction between sulfated polysaccharides and CysD on macrophages to bind AmB-loaded gelatin A nanoparticles to carboxymethylated i-carrageenan, which resulted in notably stronger antifungal activity (Aparna et al., 2018).

Wang et al. (2023) described a method involving micafungin-encapsulated conjugated polymer nanoparticles to enhance the immune response against pathogenic fungi in macrophages, aiding in the removal of intracellular infections. When exposed to near-infrared (NIR) irradiation, these nanoparticles induced photothermal destruction and facilitated the release of drugs, which exposed β-glucans on the surface of fungal conidia. This exposure allowed macrophages to identify the fungal conidia, activating the calcium (Ca^2+^)-calmodulin protein signaling pathway and the LC3-associated phagocytosis (LAP) pathway, ultimately leading to the destruction of the fungal conidia (Wang et al., 2023).

Using mannosylated chitosan oligosaccharides, Gao et al. created a mannosylated nanoparticle that contained imatinib. Imatinib is a well-known competitive inhibitor of a few tyrosine kinases, including BCR-ABL, and the platelet-derived growth factor receptors (Joensuu and Dimitrijevic, 2001). Imatinib decreased the number of M2 macrophages by blocking the STAT6 phosphorylation pathway. Chitosan oligosaccharides promoted macrophage repolarization to the M1 phenotype by activating the TLR-4 pathway. Targeting macrophages was improved by mannose motifs. In order to eradicate *C. albicans* infections, the nanotrinity may cause *in situ* remodeling of macrophages, “turning on” M1 phenotypic polarization and “turning off” M2 phenotype polarization (Gao et al., 2020).

### 3.6 Smart antifungal nanostructures

Fungal infections produce distinct microenvironments at infection sites due to the development of fungal biofilm, which are marked by changed physiological and biochemical characteristics that might be specifically targeted for treatment. Reduced pH levels, increased ROS, and enhanced enzyme activity are common in these conditions and are essential for the survival and pathogenicity of fungi. Both fungal and host tissues may sustain elevated ROS levels, which are produced in excess during infections as part of the immune system’s defense against pathogens which can exacerbate tissue damage and chronic inflammation (Tudzynski et al., 2012; Warris and Ballou, 2019). In order to facilitate fungal invasion and immune evasion, host tissues are broken down by increased activity of fungal enzymes including lipases and proteases (Wei et al., 2023). Fungal metabolism and the host’s inflammatory response frequently result in lower pH levels, which promote the growth of fungi like *C. albicans* and the creation of biofilms (Davis, 2003). In this regard, an inventive tactic is the designing of nanoparticles that release medications into the contaminated milieu in response to particular physiological cues, such as changes in pH and ROS.

A typical occurrence of fungal infections is the buildup of ROS. To successfully treat fungal infections, it is therefore promising to create nanomaterials that can react to ROS levels and release medications accordingly. When exposed to high levels of ROS, these nanomaterials can alter their solubility or structure, releasing their payloads to act directly on the infected locations and eradicate fungus. Furthermore, it has been demonstrated that materials capable of consuming ROS can efficiently reduce oxidative stress (Huang et al., 2021). To treat fungal keratitis, the researchers created and manufactured nano-based eye drops in which nanocarrier was loaded with the antifungal medication voriconazole and has glycol chitosan as the base, modified with 4-carboxyphenylboronic acid pinacol ester as the ROS-responsive group. The antifungal drug may be released when ROS combine with borate esters during inflammation or infection, causing cleavage or a change in the borate structure. In conclusion, this nano-based system may react to ROS and eradicate them via medication release and the antioxidant qualities of carriers, offering a novel approach to the management of fungal keratitis (Niu et al., 2023). According to *in vivo* data in a mouse model of fungal keratitis, the developed nanodrops showed strong retention in the cornea, high penetration through ocular barriers, and controlled drug release at low ROS concentrations.

Similarly, when compared to normal tissues, the pH values of the fungal infection microenvironment and the biofilms that form are usually lower. In a typical physiological setting, the pH-responsive antifungal nanoparticles need to be stale with little medication loss. Nonetheless, the fungicidal action of antifungal medications may be efficiently emitted from the nanoparticles in the fungal-infected microenvironment. Three types of pH-responsive processes may be distinguished based on the chemical structures of the molecules involved: amine and carboxyl groups being protonated or deprotonated; chemical bonds being broken; and supramolecular assembly or disassembly (Huang et al., 2022).

One typical method for creating pH-responsive nanoplatforms is the protonation of amine groups. The antifungal medication itraconazole was specifically loaded into pH-responsive micelles based on poly(ethylene glycol) ethyl ether methacrylate (PEGMA) and poly 2-(diethylamino) ethyl methacrylate (DEAEMA) block-copolymers of P(PEGMA-b-DEAEMA) to fight *C. albicans* biofilms. The pH-sensitive tertiary amine modules of DEAEMA were protonated in the acidic milieu of *C. albicans* biofilms, resulting in the release of itraconazole (Albayaty et al., 2020).

The formation and disintegration of supramolecular structures, where metal–polyphenol networks are well-known for their acid-triggered antibacterial qualities, provide another pH-responsive process (Guo et al., 2025; Huang et al., 2023). Furthermore, studies have used the plant immune inducer poly(salicylic acid) (PSA) and the antifungal drug tebuconazole to create directly assembled nanoparticles. Non-covalent interactions serve as the binding force between these nanoparticles which resulting into acid-responsive behavior in certain acidic microenvironments brought on by plant pathogen invasion, working in concert with the antifungal drug and plant immune inducer to provide antimicrobial effects. In addition to having a longer effective duration, stimulus-responsive co-delivery of fungicidal nanosystems also considerably decreased toxicity of tebuconazole (Huang et al., 2024).

Another crucial factor is the increased activity of certain enzymes in the microenvironment of fungal infection sites offers special prospects for the delivery of enzyme-responsive nanomedicines. For example, a crucial member of the MMP family, matrix metalloproteinase 3 (MMP-3), often referred to as stromelysin-1, is highly expressed in the infectious milieu and serves as the basis for targeted medication delivery against fungal infections. MMP-3 responsive micro-to-nano device was developed to deliver drugs precisely to treat complicated fungal diseases. This approach achieved precision drug administration by using bovine serum albumin, a natural ligand for secreted protein acidic and rich in cysteine (SPARC) in several associated target cells, as the substrate for the nanoparticles and creating microspheres via a particular MMP-3 sensitive peptide linkage (Cheng et al., 2021). The significance of the SPARC-mediated uptake mechanism was demonstrated by the greatly increased penetration of these nanoparticles in the infected lung and brain, as well as the overlap between SPARC and the nanoparticles distribution in mice models infected by *C. neoformans*.

Another important elevated enzyme that is released by different fungi in the fungal-infected microenvironment is lipase. Drug delivery has been investigated using poly(ethylene glycol)-poly-(ε-caprolactone) (PEG-PCL) polymers, which are renowned for their amphiphilicity, superior biocompatibility, and sensitivity to lipase degradation. Dong et al. created polymeric nanostructures by loading fluconazole and diketopyrrolopyrrole onto PGL. When diketopyrrolopyrrole is activated by light, it may produce heat and ROS. At the infection site, lipase generated by *C. albicans* may break down PGL polymer and cause FLU to be released. With outstanding antifungal qualities, this system allowed the simultaneous administration of antibiotic treatment, photodynamic therapy, and photothermal therapy (Yang et al., 2019).

Additionally, fungi have the ability to release hyaluronidase, which hydrolyzes hyaluronic acid (HA) specifically. *C. albicans* is usually the cause of the common fungal vaginal inflammatory condition known as candidial vaginitis. For instance, HA may be broken down by the enzyme Hyaluronidase, which is released by bacteria and *C. albicans*. A HA hydrogel called rGO@FeS_2_/*Lactobacillus*@HA was created by Wei et al. to cure *Candida* vaginitis-infected mice and lower recurrence. Lactobacilli and rGO@FeS_2_ nanozymes were released locally when HA underwent enzymatic breakdown in the vaginal microenvironment. The vaginal microenvironment was normalized and the pH was lowered to 4 to 4.5 as a result of Lactobacilli’s fermentation and production of lactic acid. However, Lactobacillus-produced H_2_O_2_ might be catalyzed by the rGO@FeS_2_ nanozymes to produce a significant quantity of OH, which would kill *C. albicans* (Wei et al., 2023).

### 3.7 Cellular targeted antifungal nanostructures

To increase the efficacy of antifungal treatments, it is essential to penetrate tissue-specific barriers such the skin, cornea, and blood–brain barrier (BBB). An efficient method for getting past these obstacles and directly to the infection sites is provided by targeted nanotechnology. First, drug entry is impeded by the thick barriers formed by the fungal cell walls and extracellular polysaccharides of biofilms. A key factor in fungal medication resistance is the polysaccharides found in their cell walls, such as β-1,3-glucan (Lipke and Ovalle, 1998).

He et al. suggested a method that entailed breaking down extracellular polysaccharides, specifically β-1,3-glucan, in order to enhance the sensitivity of antifungal techniques and penetrate the fungal cell wall and extracellular polysaccharide barriers. They created an integrated nanosystem that can break down the extracellular polysaccharides found in cell walls and biofilms by combining lyticase and gallium ions. By releasing gallium ions, this nanosystem eliminated mature biofilms and *C. albicans*. In a fungal keratitis animal model, this approach produced adequate therapeutic results, demonstrating the possibility of overcoming fungal resistance pathways by interfering with antioxidant-, exopolysaccharide-, iron-ion-utilization-, biofilm-development-, and virulence-related genes (He et al., 2022).

Photothermal therapy (PTT), praised for its strong control, simplicity of use, biosafety, and dependability, is becoming more and more acknowledged as a potential anti-infective therapeutic approach. Because of NIR lasers’ exceptional tissue penetration capabilities, NIR laser-powered nanomotors are becoming a premium drug delivery method that can deliver therapeutic compounds to deep tissue areas. A miconazole nitrate-loaded parachute-like nanomotor (MN) powered by an NIR laser was also developed by Ji et al. (2021). Skin mycosis, dermatophytosis, and oropharyngeal candidiasis were among the fungal disorders that were treated with MN, a broad-spectrum antifungal azole. Under NIR irradiation, nanomotors with a parachute-like Janus structure may create a temperature gradient that would cause autonomous motion via the resulting self-thermophoresis, improving MN absorption and biofilm adherence. Drug penetration through the skin into the infection site was made a promising strategy by this NIR-powered nanomotor.

Using hyaluronic acid loaded with CuS nanoparticles and the antimicrobial peptide PAF-26, Zhao and colleagues created a new biodegradable microneedle patch for the treatment of deep cutaneous fungal infection (DCFI). Following epidermal penetration, microneedle tips disintegrated gradually, releasing PAF-26 and CuS nanoparticles to eradicate fungus. To target fungi, CuS nanoparticles catalyze H_2_O_2_ to create ROS, whereas PAF-26 directly damages fungal cell envelopes. ROS quickly enter the fungal body once PAF-26 breaks down the fungal cell membranes, increasing the antifungal action. An essential approach to treating DCFI was made possible by microneedle technology, which improved medication transdermal efficiency, decreased administration frequency, and prevented resistance problems (Wang et al., 2023).

Shi et al. (2024) created a multi-enzyme mimicking copper-proanthocyanidins nanozyme-thixotropic anionic hydrogel coating (CuPC’s) for the treatment of fungal keratitis in order to improve drug delivery and prolong the duration of drug retention on the ocular surface. This was accomplished by a Schiff base reaction between amino-functionalized hyaluronic acid and a self-synthesized polyaldehyde oligomer. In reaction to changes in stress or strain, the thixotropic hydrogel may change its viscosity or flow characteristics. Voriconazole’s retention duration and permeability were significantly extended by this hydrogel coating, allowing for a lower dose to achieve therapeutically efficacious levels. Additionally, by using a HA derivative to stimulate cell proliferation, this hydrogel coating aided in the repair of corneal wounds. Furthermore, CuPC’s catalase-like and superoxide dismutase-like properties helped to fight ROS, improving the therapeutic effectiveness in the treatment of fungal keratitis. A mouse model of fungal keratitis caused by *F. solani* was then used to examine the impact of the hydrogel on treating the condition. It demonstrated antifungal activity by scavenging ROS through the catalase-like and superoxide dismutase-like activities.

### 3.8 Combination strategies

Combination therapy presents an exciting opportunity to tackle the increasing challenge of fungal resistance effectively. By leveraging advances in nanotechnology, researchers can combine multiple antifungal treatments into a single nanoparticle, significantly enhancing treatment efficacy and helping to delay the onset of drug resistance through diversified antifungal mechanisms. Sun et al. developed CuO nanoparticle composites with polylysine–alginate nanogels to address *A. alternata* infections in plants. Their findings illustrate the potential for these composites to provide synergistic fungicidal effects through the combined action of polylysine and copper ions (Zhu et al., 2022).

## 4 Conclusion

Fungal infections pose a significant challenge in clinical treatment and have emerged as a pressing public health issue. While existing antifungal medications such as polyenes, azoles, echinocandins, and pyrimidine analogs have proven effective, their success is increasingly compromised by the rapid development of resistance. This situation emphasizes the importance of addressing the shortcomings of current antifungal treatments, particularly given the rising number of fungal infections, high mortality rates, and limited options available to healthcare providers. A promising approach to tackle these challenges lies in the innovative use of nanotechnology. By utilizing various nanotechnology processes, we can potentially enhance the efficacy of antifungal therapies, offering new avenues for treatment and improving patient outcomes.

Numerous significant issues with conventional antifungal therapies have been shown to be resolved using nanotechnology. Although nanotechnology holds immense promise for antifungal treatment, numerous obstacles remain. The majority of research on antifungal nanoparticles is now restricted to *in vitro* tests. It is difficult to create animal models of fungal infections that closely mimic human illnesses, which limits the assessment and improvement of nanoparticles in preclinical research. There is currently a lack of information regarding the long-term biosafety, metabolic routes, and biodistribution of nanomedicines. Additional research is also needed to assess the potential toxicity, long-term biological consequences, and biocompatibility of nanomedicines.

Furthermore, their exact design with high antifungal efficacy is limited by the incomplete understanding of the mechanisms underlying the interaction between nanoparticles and fungal cells. In summary, nanotechnology offers a promising approach to antifungal treatment. With continuous advancements and a deeper understanding of how fungal infections work, nanotechnology is expected to play an increasingly important role in antifungal therapies in the future.

High-throughput screening technologies play a pivotal role in uncovering strong antifungal drugs from extensive libraries of small compounds and polymers, significantly enhancing the speed and efficiency of antifungal therapeutic development. By incorporating nanotechnology into this process, we can further amplify the efficacy of these newly discovered antifungal medications. Nanotechnology facilitates the precise delivery of antifungals directly to the site of infection and optimizes release profiles for sustained therapeutic effects. As a result, the synergy between high-throughput screening and nanotechnology not only promotes the effectiveness and safety of new antifungal drugs but also accelerates their discovery and development timeline. This innovative approach holds great promise for transforming the antifungal therapy landscape, ensuring that more potent antifungal treatments reach the market in a timely manner.

## References

[B1] Ahmad T. Wani I. A. Lone I. H. Ganguly A. Manzoor N. Ahmad A. (2013). Antifungal activity of gold nanoparticles prepared by solvothermal method. Mater. Res. Bull. 48 (1), 12–20. 10.1016/j.materresbull.2012.09.069

[B2] Al-thabaiti S. A. Khan Z. Manzoor N. (2015). Biosynthesis of silver nanoparticles and its antibacterial and antifungal activities towards Gram-positive, Gram-negative bacterial strains and different species of Candida fungus. Bioprocess Biosyst. Eng. 38 (9), 1773–1781. 26017756 10.1007/s00449-015-1418-3

[B3] Albayaty Y. N. Thomas N. Ramírez-García P. D. Davis T. P. Quinn J. F. Whittaker M. R. (2020). pH-Responsive copolymer micelles to enhance itraconazole efficacy against Candida albicans biofilms. J. Mater Chem. B 8 (8), 1672–1681. 10.1039/c9tb02586c 32016213

[B4] Alli Y. A. Ejeromedoghene O. Oladipo A. Adewuyi S. Amolegbe S. A. Anuar H. (2022). Compressed hydrogen-induced synthesis of Quaternary trimethyl chitosan-silver nanoparticles with dual antibacterial and antifungal activities. ACS Appl. Bio Mater. 5 (11), 5240–5254. 10.1021/acsabm.2c00670 36270024

[B5] Alli Y. A. Ejeromedoghene O. Oladoye P. O. Bamisaye A. Oladoyinbo F. O. Adewuyi S. (2024). Hydrogen-assisted green synthesis of trimethyl chitosan gold nanoparticles. Kuwait J. Sci. 51 (2), 100162. 10.1016/j.kjs.2023.12.002

[B6] Ambati S. Ellis E. C. Lin J. Lin X. Lewis Z. A. Meagher R. B. (2019). Dectin-2-Targeted antifungal liposomes exhibit enhanced efficacy. mSphere 4 (5), e00715-19. 10.1128/mSphere.00715-19 31666315 PMC6821932

[B7] Ambati S. Ferarro A. R. Kang S. E. Lin J. Lin X. Momany M. (2019). Dectin-1-Targeted antifungal liposomes exhibit enhanced efficacy. mSphere 4 (1), e00025-19. 10.1128/mSphere.00025-19 30760610 PMC6374590

[B8] Aparna V. Melge A. R. Rajan V. K. Biswas R. Jayakumar R. Gopi Mohan C. (2018). Carboxymethylated ɩ-carrageenan conjugated amphotericin B loaded gelatin nanoparticles for treating intracellular Candida glabrata infections. Int. J. Biol. Macromol. 110, 140–149. 10.1016/j.ijbiomac.2017.11.126 29169943

[B9] Araujo V. H. S. Duarte J. L. Carvalho G. C. Silvestre A. L. P. Fonseca-Santos B. Marena G. D. (2020). Nanosystems against candidiasis: a review of studies performed over the last two decades. Crit. Rev. Microbiol. 46, 508–547. 10.1080/1040841X.2020.1803208 32795108

[B10] Bongomin F. Gago S. Oladele R. O. Denning D. W. (2017). Global and multi-national prevalence of fungal diseases-estimate precision. J. Fungi (Basel) 3 (4), 57. 10.3390/jof3040057 29371573 PMC5753159

[B11] Brown G. D. Denning D. W. Gow N. W. Levitz S. M. Netea M. G. White T. C. (2012). Hidden killers: human fungal infections. Sci. Transl. Med. 4, 165rv13. 10.1126/scitranslmed.3004404 23253612

[B12] Campoy S. Adrio J. L. (2017). Antifungals. Biochem. Pharmacol. 133, 86–96. 10.1016/j.bcp.2016.11.019 27884742

[B13] Cao V. D. Nguyen P. P. Khuong V. Q. Nguyen C. K. Nguyen X. C. Dang C. H. (2014). Ultrafine copper nanoparticles exhibiting a powerful antifungal/killing activity against Corticium salmonicolor. Bull. Korean Chem. Soc. 35 (9), 2645–2648. 10.5012/bkcs.2014.35.9.2645

[B14] Carolus H. Pierson S. Lagrou K. Van Dijck P. (2020). Amphotericin B and other polyenes-discovery, clinical use, mode of action and drug resistance. J. Fungi (Basel) 6 (4), 321. 10.3390/jof6040321 33261213 PMC7724567

[B15] Chen S. C. Slavin M. A. Sorrell T. C. (2011). Echinocandin antifungal drugs in fungal infections: a comparison. Drugs 71 (1), 11–41. 10.2165/11585270-000000000-00000 21175238

[B16] Chen H. Geng X. Ning Q. Shi L. Zhang N. He S. (2024). Biophilic positive carbon dot exerts antifungal activity and augments corneal permeation for Fungal Keratitis. Nano Lett. 24 (13), 4044–4053. 10.1021/acs.nanolett.4c01042 38517749

[B17] Chen L. Shao Z. Zhang Z. Teng W. Mou H. Jin X. (2024). An On-Demand collaborative innate-adaptive immune response to infection treatment. Adv. Mater 36 (15), e2304774. 10.1002/adma.202304774 37523329

[B18] Cheng L. Niu M. M. Yan T. Ma Z. Huang K. Yang L. (2021). Bioresponsive micro-to-nano albumin-based systems for targeted drug delivery against complex fungal infections. Acta Pharm. Sin. B 11 (10), 3220–3230. 10.1016/j.apsb.2021.04.020 34729311 PMC8546853

[B19] Cioffi N. Torsi L. Ditaranto N. Sabbatini L. Zambonin P. G. Tantillo G. (2004). Antifungal activity of polymer-based copper nanocomposite coatings. Appl. Phys. Lett. 85 (12), 2417–2419. 10.1063/1.1794381

[B20] Cioffi N. Torsi L. Ditaranto N. Tantillo G. Ghibelli L. Sabbatini L. (2005). Copper nanoparticle/polymer composites with antifungal and bacteriostatic properties. Chem. Mater. 17 (21), 5255–5262. 10.1021/cm0505244

[B21] Coelho R. A. Almeida-Silva F. Figueiredo-Carvalho M. H. G. Rabello V. B. S. de Souza G. R. Lourenço MCDS (2024). Comparison of the antifungal activity of the pyrimidine analogs flucytosine and carmofur against human-pathogenic dematiaceous fungi. Med. Mycol. 62 (4), myae029. 10.1093/mmy/myae029 38533658 PMC11008743

[B22] Como Jackson A. Dismukes William E. (1994). Oral azole drugs as systemic antifungal therapy. N. Engl. J. Med. 330, 263–272. 10.1056/NEJM199401273300407 8272088

[B23] Crawford F. Hollis S. (2007). Topical treatments for fungal infections of the skin and nails of the foot. Cochrane Database Syst. Rev. 3, CD001434. 10.1002/14651858.CD001434.pub2 17636672 PMC7073424

[B24] Ćurić A. Möschwitzer J. P. Fricker G. (2017). Development and characterization of novel highly-loaded itraconazole poly(butyl cyanoacrylate) polymeric nanoparticles. Eur. J. Pharm. Biopharm. 114, 175–185. 10.1016/j.ejpb.2017.01.014 28159723

[B25] Dananjaya S. H. S. Edirisinghe S. L. Thao N. T. T. Kumar R. S. Wijerathna HMSM Mudiyanselage A. Y. (2020). Succinyl chitosan gold nanocomposite: preparation, characterization, *in vitro* and *in vivo* anticandidal activity. Int. J. Biol. Macromol. 165 (Pt A), 63–70. 10.1016/j.ijbiomac.2020.09.126 32971172

[B26] Dananjaya S. Thao N. T. Wijerathna H. Lee J. Edussuriya M. Choi D. (2020). *In vitro* and *in vivo* anticandidal efficacy of green synthesized gold nanoparticles using *Spirulina maxima* polysaccharide. Process Biochem. 92, 138–148. 10.1016/j.procbio.2020.03.003

[B27] Davis D. (2003). Adaptation to environmental pH in *Candida albicans* and its relation to pathogenesis. Curr. Genet. 44 (1), 1–7. 10.1007/s00294-003-0415-2 12819929

[B28] Denning D. W. (2024). Global incidence and mortality of severe fungal disease. Lancet Infect. Dis. 24 (7), e428–e438. 10.1016/S1473-3099(23)00692-8 38224705

[B29] Du W. Gao Y. Liu L. Sai S. Ding C. (2021). Striking back against fungal infections: the utilization of nanosystems for antifungal strategies. Int. J. Mol. Sci. 22, 10104. 10.3390/ijms221810104 34576268 PMC8466259

[B30] Erdogar N. Akk n S. Bilensoy E. (2018). Nanocapsules for drug delivery: an updated review of the last decade. Recent pat. Drug Deliv. Formul. 12, 252. 10.2174/1872211313666190123153711 30674269

[B31] Fisher M. C. Alastruey-Izquierdo A. Berman J. Bicanic T. Bignell E. M. Bowyer P. (2022). Tackling the emerging threat of antifungal resistance to human health. Nat. Rev. Microbiol. 20 (9), 557–571. 10.1038/s41579-022-00720-1 35352028 PMC8962932

[B32] Gao Q. Zhang J. Chen C. Chen M. Sun P. Du W. (2020). *In situ* mannosylated nanotrinity-mediated macrophage remodeling combats *Candida albicans* infection. ACS Nano 14, 3980–3990. 10.1021/acsnano.9b07896 32167741

[B33] Gao Y. Zhou Z. Tang G. Tian Y. Zhang X. Huang Y. (2024). Facile fabrication of a fungicide and plant immune inducer co-delivery nanosystem for enhanced control efficacy against plant disease. Chem. Eng. J. 482, 148817. 10.1016/j.cej.2024.148817

[B34] Garibotto F. M. Garro A. D. Masman M. F. Rodríguez A. M. Luiten P. G. M. Raimondi M. (2010). New small-size peptides possessing antifungal activity. Bioorg Med. Chem. 18, 158–167. 10.1016/j.bmc.2009.11.009 19959366

[B35] Gavaldà Jmartín M. López P. Gomis X. Ramírez J. Rodríguez D. Len O. (2005). Efficacy of nebulized liposomal amphotericin B in treatment of experimental pulmonary aspergillosis. Antimicrob. Agents Chemother. 49, 3028–3030. 10.1128/AAC.49.7.3028-3030.2005 15980392 PMC1168712

[B36] Golipour F. Habibipour R. Moradihaghgou L. (2019). Investigating effects of superparamagnetic iron oxide nanoparticles on *Candida albicans* biofilm formation. Med. Lab. J. 13 (6), 44–50. 10.29252/mlj.13.6.44

[B37] Guo Q. Liu Y. Huang Y. Hu G. Tang G. Zhang X. (2025). Nanocapsules bearing imide polymer as wall material for pH-responsive and synergistic fungicidal activity. Chem. Eng. J. 10.1016/j.cej.2025.166144

[B38] Gupta R. Xie H. (2018). Nanoparticles in daily life: applications, toxicity and regulations. J. Environ. Pathol. Toxicol. Oncol. 37, 209–230. 10.1615/JEnvironPatholToxicolOncol.2018026009 30317972 PMC6192267

[B39] Han H. Bártolo R. Li J. Shahbazi M. A. Santos H. A. (2022). Biomimetic platelet membrane-coated nanoparticles for targeted therapy. Eur. J. Pharm. Biopharm. 172, 1–15. 10.1016/j.ejpb.2022.01.004 35074554

[B40] Hashimoto S. (2009). Micafungin: a sulfated echinocandin. J. Antibiot. (Tokyo) 62 (1), 27–35. 10.1038/ja.2008.3 19132058

[B41] He J. Ye Y. Zhang D. Yao K. Zhou M. (2022). Visualized Gallium/lyticase-integrated antifungal strategy for Fungal Keratitis treatment. Adv. Mater. 34, 2206437. 10.1002/adma.202206437 36177690

[B42] Huang X. He D. Pan Z. Luo G. Deng J. (2021). Reactive-oxygen-species-scavenging nanomaterials for resolving inflammation. Mater Today Bio 11, 100124. 10.1016/j.mtbio.2021.100124 34458716 PMC8379340

[B43] Huang Y. Zou L. Wang J. Jin Q. Ji J. (2022). Stimuli-responsive nanoplatforms for antibacterial applications. Wiley Interdiscip. Rev. Nanomed Nanobiotechnol 14 (3), e1775. 10.1002/wnan.1775 35142071

[B44] Huang Y. Chen Y. Lu Z. Yu B. Zou L. Song X. (2023). Facile synthesis of self-targeted Zn^2+^ -Gallic acid nanoflowers for specific adhesion and elimination of gram-positive bacteria. Small 19 (43), e2302578. 10.1002/smll.202302578 37376855

[B45] Huang Y. Wang H. Tang G. Zhou Z. Zhang X. Liu Y. (2024). Fabrication of pH-responsive nanoparticles for co-delivery of fungicide and salicylic acid with synergistic antifungal activity. J. Clean. Prod. 451, 142093. 10.1016/j.jclepro.2024.142093

[B46] Ji X. Yang H. Liu W. Ma Y. Wu J. Zong X. (2021). Multifunctional parachute-like nanomotors for enhanced skin penetration and synergistic antifungal therapy. ACS Nano 15 (9), 14218–14228. 10.1021/acsnano.1c01379 34435494

[B47] Jiao L. Seow J. Y. R. Skinner W. S. Wang Z. U. Jiang H.-L. (2019). Metal–organic frameworks: structures and functional applications. Mater. Today 27, 43–68. 10.1016/j.mattod.2018.10.038

[B48] Joensuu H. Dimitrijevic S. (2001). Tyrosine kinase inhibitor imatinib (STI571) as an anticancer agent for solid tumours. Ann. Med. 33 (7), 451–455. 10.3109/07853890109002093 11680792

[B49] Kang X. Kirui A. Muszyński A. Widanage M. C. D. Chen A. Azadi P. (2018). Molecular architecture of fungal cell walls revealed by solid-state NMR. Nat. Commun. 9 (1), 2747. 10.1038/s41467-018-05199-0 30013106 PMC6048167

[B50] Kreuter J. (2007). Nanoparticles--a historical perspective. Int. J. Pharm. 331 (1), 1–10. 10.1016/j.ijpharm.2006.10.021 17110063

[B51] Kumar S. Kaur P. Bernela M. Rani R. Thakur R. (2016). Ketoconazole encapsulated in chitosan-gellan gum nanocomplexes exhibits prolonged antifungal activity. Int. J. Biol. Macromol. 93, 988–994. 10.1016/j.ijbiomac.2016.09.042 27659003

[B52] Lewis R. E. (2010). Aspergillosis: from diagnosis to prevention. Dordrecht: Springer, 281–305.

[B53] Lewis J. S. Wiederhold N. P. Hakki M. Thompson G. R. (2022). New perspectives on antimicrobial agents: isavuconazole. Antimicrob. Agents Chemother. 66 (9), e0017722. 10.1128/aac.00177-22 35969068 PMC9487460

[B54] Li C. Wang X. Chen F. Zhang C. Zhi X. Wang K. (2013). The antifungal activity of graphene oxide–silver nanocomposites. Biomaterials 34 (15), 3882–3890. 10.1016/j.biomaterials.2013.02.001 23465487

[B55] Lin Y. Yin Q. Tian D. Yang X. Liu S. Sun X. (2023). Vaginal epithelial cell membrane-based phototherapeutic decoy confers a “Three-in-One” strategy to treat against intravaginal infection of *Candida albicans* . ACS Nano 17 (13), 12160–12175. 10.1021/acsnano.2c12644 37200053

[B56] Lipke P. N. Ovalle R. (1998). Cell wall architecture in yeast: new structure and new challenges. J. Bacteriol. 180 (15), 3735–3740. 10.1128/JB.180.15.3735-3740.1998 9683465 PMC107352

[B57] Liu X. Tao Y. Zhang L. Liu Y. Shi D. Wang J. (2025). Caged-hypocrellin mediated photodynamic therapy induces chromatin remodeling and disrupts mitochondrial energy metabolism in multidrug-resistant *Candida auris* . Redox Biol. 85, 103708. 10.1016/j.redox.2025.103708 40544603 PMC12221887

[B58] Liu X. Guo S. Wang J. Zhang L. Lin Y. Liu Y. (2025). Caged-hypocrellin-mediated antimicrobial photodynamic therapy as a dual-action strategy for fungal clearance and immune response regulation in drug-resistant *Candida auris* wound infections. J. Am. Acad. Dermatol. 10.1016/j.jaad.2025.08.040 40850383

[B59] Mallmann E. J. J. Cunha F. A. Castro B. N. Maciel A. M. Menezes E. A. Fechine P. B. A. (2015). Antifungal activity of silver nanoparticles obtained by green synthesis. Rev. Inst. Med. Trop. São Paulo 57 (2), 165–167. 10.1590/S0036-46652015000200011 25923897 PMC4435016

[B60] Mohd-Assaad N. McDonald B. A. Croll D. (2016). Multilocus resistance evolution to azole fungicides in fungal plant pathogen populations. Mol. Ecol. 25, 6124–6142. 10.1111/mec.13916 27859799

[B61] Mohr J. Johnson M. Cooper T. Lewis J. S. Ostrosky-Zeichner L. (2008). Current options in antifungal pharmacotherapy. Pharmacother. J. Hum. Pharmacol. Drug Ther. 28, 614–645. 10.1592/phco.28.5.614 18447660

[B62] Monteiro D. Silva S. Negri M. Gorup L. De Camargo E. Oliveira R. (2012). Silver nanoparticles: influence of stabilizing agent and diameter on antifungal activity against *Candida albicans* and *Candida glabrata* biofilms. Lett. Appl. Microbiol. 54 (5), 383–391. 10.1111/j.1472-765X.2012.03219.x 22313289

[B63] Mota Fernandes C. Dasilva D. Haranahalli K. McCarthy J. B. Mallamo J. Ojima I. (2021). The future of antifungal drug therapy: novel compounds and targets. Antimicrob. Agents Chemother. 65 (2), e01719-20. 10.1128/AAC.01719-20 33229427 PMC7848987

[B64] Mu L. Feng S. S. (2003). A novel controlled release formulation for the anticancer drug paclitaxel (Taxol®): PLGA nanoparticles containing vitamin E TPGS. J. Control Release 86, 33–48. 10.1016/s0168-3659(02)00320-6 12490371

[B65] Munin A. Edwards-Lévy F. (2011). Encapsulation of natural polyphenolic compounds; a review. Pharmaceutics 3 (4), 793–829. 10.3390/pharmaceutics3040793 24309309 PMC3857059

[B66] Nami S. Aghebati-Maleki A. Aghebati-Maleki L. (2021). Current applications and prospects of nanoparticles for antifungal drug delivery. EXCLI J. 20, 562–584. 10.17179/excli2020-3068 33883983 PMC8056051

[B67] Negi P. Singh A. Pundir S. Parashar A. Upadhyay N. Agarwal S. (2023). Essential oil and nanocarrier-based formulations approaches for vaginal candidiasis. Ther. Deliv. 14, 207–225. 10.4155/tde-2022-0058 37191049

[B68] Niu P. Wu Y. Zeng F. Zhang S. Liu S. Gao H. (2023). Development of nanodrug-based eye drops with good penetration properties and ROS responsiveness for controllable release to treat fungal keratitis. NPG Asia Mater. 15, 31. 10.1038/s41427-023-00478-9

[B69] Panwar R. Pemmaraju S. C. Sharma A. K. Pruthi V. (2016). Efficacy of ferulic acid encapsulated chitosan nanoparticles against *Candida albicans* biofilm. Microb. Pathog. 95, 21–31. 10.1016/j.micpath.2016.02.007 26930164

[B70] Peer D. Karp J. M. Hong S. Farokhzad O. C. Margalit R. Langer R. (2007). Nanocarriers as an emerging platform for cancer therapy. Nat. Nanotechnol. 2, 751–760. 10.1038/nnano.2007.387 18654426

[B71] Perlin D. S. Rautemaa-Richardson R. Alastruey-Izquierdo A. (2017). The global problem of antifungal resistance: prevalence, mechanisms, and management. Lancet Infect. Dis. 17 (12), e383–e392. 10.1016/S1473-3099(17)30316-X 28774698

[B72] Rahisuddin A.-T. S. A. Khan Z. Manzoor N. (2015). Biosynthesis of silver nanoparticles and its antibacterial and antifungal activities towards Gram-positive, Gram-negative bacterial strains and different species of Candida fungus. Bioprocess Biosyst. Eng. 38 (9), 1773–1781. 10.1007/s00449-015-1418-3 26017756

[B73] Raut J. S. Shinde R. B. Chauhan N. M. Karuppayil S. M. (2014). Phenylpropanoids of plant origin as inhibitors of biofilm formation by *Candida albicans* . J. Microbiol. Biotechnol. 24 (9), 1216–1225. 10.4014/jmb.1402.02056 24851813

[B74] Roemer T. Krysan D. J. (2014). Antifungal drug development: challenges, unmet clinical needs, and new approaches. Cold Spring Harb. Perspect. Med. 4, a019703. 10.1101/cshperspect.a019703 24789878 PMC3996373

[B75] Rokas A. (2022). Evolution of the human pathogenic lifestyle in fungi. Nat. Microbiol. 7, 607–619. 10.1038/s41564-022-01112-0 35508719 PMC9097544

[B76] Roy B. K. Tahmid I. Rashid T. U. (2021). Chitosan-based materials for supercapacitor applications: a review. J. Mater. Chem. A 9, 17592–17642. 10.1039/D1TA02997E

[B77] Sebti I. Martial-Gros A. Carnet-Pantiez A. Grelier S. Coma V. (2005). Chitosan polymer as bioactive coating and film against *Aspergillus niger* contamination. J. Food Sci. 70, M100–M104. 10.1111/j.1365-2621.2005.tb07098.x

[B78] Shi D. Qi X. Ma L. Zhao L. Dou S. Wang Y. (2024). Fabrication of nanozyme-thixotropic anionic hydrogel coating with multi-enzyme-mimicking activity for the treatment of fungal keratitis. Chem. Eng. J. 486, 150264. 10.1016/j.cej.2024.150264

[B80] Su L. Li Y. Liu Y. Ma R. Liu Y. Huang F. (2020). Antifungal-inbuilt Metal–organic-frameworks eradicate *Candida albicans* biofilms. Adv. Funct. Mater. 30, 2000537. 10.1002/adfm.202000537

[B81] Swain S. Barik S. Behera T. Nayak S. Sahoo S. Mishra S. (2016). Green synthesis of gold nanoparticles using root and leaf extracts of Vetiveria zizanioides and Cannabis sativa and its antifungal activities. BioNanoScience 6 (3), 205–213. 10.1007/s12668-016-0208-y

[B82] Tang Y. Wu S. Lin J. Cheng L. Zhou J. Xie J. (2018). Nanoparticles targeted against cryptococcal pneumonia by interactions between chitosan and its peptide ligand. Nano Lett. 18 (10), 6207–6213. 10.1021/acs.nanolett.8b02229 30260652

[B83] Tang S. Chen J. Cannon J. Cao Z. Baker J. R. Jr Wang S. H. (2021). Dendrimer-based posaconazole nanoplatform for antifungal therapy. Drug Deliv. 28 (1), 2150–2159. 10.1080/10717544.2021.1986605 34617850 PMC8510609

[B84] Tantubay S. Mukhopadhyay S. K. Kalita H. Konar S. Dey S. Pathak A. (2015). Carboxymethylated chitosan-stabilized copper nanoparticles: a promise to contribute a potent antifungal and antibacterial agent. J. Nanopart. Res. 17 (6), 243. 10.1007/s11051-015-3047-9

[B85] Tudzynski P. Heller J. Siegmund U. (2012). Reactive oxygen species generation in fungal development and pathogenesis. Curr. Opin. Microbiol. 15 (6), 653–659. 10.1016/j.mib.2012.10.002 23123514

[B86] Tufa T. B. Denning D. W. (2019). The burden of fungal infections in Ethiopia. J. Fungi 5, 109. 10.3390/jof5040109 31771096 PMC6958437

[B87] Vazquez-Muñoz R. Avalos-Borja M. Castro-Longoria E. (2014). Ultrastructural analysis of Candida albicans when exposed to silver nanoparticles. PLoS One 9 (10), e108876. 10.1371/journal.pone.0108876 25290909 PMC4188582

[B88] Vermes A. Guchelaar H. J. Dankert J. (2000). Flucytosine: a review of its pharmacology, clinical indications, pharmacokinetics, toxicity and drug interactions. J. Antimicrob. Chemother. 46 (2), 171–179. 10.1093/jac/46.2.171 10933638

[B89] Voltan A. Quindós G. Alarcón K. Fusco-Almeida A. M. Mendes-Giannini M. J. S. Chorilli M. (2016). Fungal diseases: could nanostructured drug delivery systems be a novel paradigm for therapy? Int. J. Nanomed 11, 3715–3730. 10.2147/IJN.S93105 27540288 PMC4982498

[B90] Vyas S. P. Katare Y. K. Mishra V. Sihorkar V. (2000). Ligand directed macrophage targeting of amphotericin B loaded liposomes. Int. J. Pharm. 210 (1-2), 1–14. 10.1016/s0378-5173(00)00522-6 11163983

[B92] Wang Z. Qu S. Gao D. Shao Q. Nie C. Xing C. (2023). A strategy of On-Demand immune activation for antifungal treatment using near-infrared responsive conjugated polymer nanoparticles. Nano Lett. 23, 326–335. 10.1021/acs.nanolett.2c04484 36548213

[B93] Wang L. Gui Y. Li K. Tao W. Li C. Qiu J. (2024). Biomimetic and multifunctional nanocomposites for precision fungi theranostics. Biomaterials 308, 122561. 10.1016/j.biomaterials.2024.122561 38603827

[B94] Wani I. A. Ahmad T. Manzoor N. (2013). Size and shape dependant antifungal activity of gold nanoparticles: a case study of Candida. Colloids Surf. B Biointerfaces 101, 162–170. 10.1016/j.colsurfb.2012.06.005 22796787

[B95] Warris A. Ballou E. R. (2019). Oxidative responses and fungal infection biology. Semin. Cell Dev. Biol. 89, 34–46. 10.1016/j.semcdb.2018.03.004 29522807

[B96] Wei Y. Chen S. Kowalczyk B. Huda S. Gray T. P. Grzybowski B. A. (2010). Synthesis of stable, low-dispersity copper nanoparticles and nanorods and their antifungal and catalytic properties. J. Phys. Chem. C 114 (37), 15612–15616. 10.1021/jp1055683

[B97] Wei G. Liu Q. Wang X. Zhou Z. Zhao X. Zhou W. (2023). A probiotic nanozyme hydrogel regulates vaginal microenvironment for *Candida* vaginitis therapy. Sci. Adv. 9 (20), eadg0949. 10.1126/sciadv.adg0949 37196095 PMC10191424

[B98] Wu S. Guo W. Li B. Zhou H. Meng H. Sun J. (2023). Progress of polymer-based strategies in fungal disease management: designed for different roles. Front. Cell Infect. Microbiol. 13, 1142029. 10.3389/fcimb.2023.1142029 37033476 PMC10073610

[B99] Xie J. Shen Q. Huang K. Zheng T. Cheng L. Zhang Z. (2019). Oriented assembly of cell-mimicking nanoparticles *via* a molecular affinity strategy for targeted drug delivery. ACS Nano 13 (5), 5268–5277. 10.1021/acsnano.8b09681 31022341

[B100] Yang D. Lv X. Xue L. Yang N. Hu Y. Weng L. (2019). A lipase-responsive antifungal nanoplatform for synergistic photodynamic/photothermal/pharmaco-therapy of azole-resistant Candida albicans infections. Chem. Commun. 55, 15145–15148. 10.1039/c9cc08463k 31790115

[B101] Ye Y. He J. Wang H. Li W. Wang Q. Luo C. (2022). Cell wall destruction and internal Cascade synergistic antifungal strategy for fungal keratitis. ACS Nano 16 (11), 18729–18745. 10.1021/acsnano.2c07444 36278973

[B102] Zhu X. Ma X. Gao C. Mu Y. Pei Y. Liu C. (2022). Fabrication of CuO nanoparticles composite ε-polylysine-alginate nanogel for high-efficiency management of *Alternaria alternate* . Int. J. Biol. Macromol. 223, 1208–1222. 10.1016/j.ijbiomac.2022.11.072 36375663

[B103] Zhu X. Chen Y. Yu D. Fang W. Liao W. Pan W. (2023). Progress in the application of nanoparticles for the treatment of fungal infections: a review. Mycology 15 (1), 1–16. 10.1080/21501203.2023.2285764 38558835 PMC10977003

